# Novel Plasma Proteomic Biomarkers for Early Identification of Induction Chemotherapy Beneficiaries in Locoregionally Advanced Nasopharyngeal Carcinoma

**DOI:** 10.3389/fonc.2022.889516

**Published:** 2022-06-30

**Authors:** Shan-Qiang Zhang, Su-Ming Pan, Shu-Zhen Lai, Hui-Jing Situ, Jun Liu, Wen-Jie Dai, Si-Xian Liang, Li-Qing Zhou, Qi-Qi Lu, Pei-Feng Ke, Fan Zhang, Hai-Bin Chen, Ji-Cheng Li

**Affiliations:** ^1^ Medical Research Center, Yuebei People’s Hospital, Shantou University Medical College, Shaoguan, China; ^2^ Department of Radiation Oncology, Yuebei People’s Hospital, Shantou University Medical College, Shaoguan, China; ^3^ Department of Histology and Embryology, Shantou University Medical College, Shantou, China; ^4^ Institute of Cell Biology, Zhejiang University School of Medicine, Hangzhou, China

**Keywords:** nasopharyngeal carcinoma, induction chemotherapy, efficacy prediction, proteomics, biomarker identification

## Abstract

**Background:**

Induction chemotherapy (IC) can alleviate locoregionally advanced nasopharyngeal carcinoma (LA-NPC), but effectiveness differs between patients, toxicity is problematic, and effective blood-based IC efficacy predictors are lacking. Here, we aimed to identify biomarkers for early identification of IC beneficiaries.

**Methods:**

Sixty-four pairs of matched plasma samples collected before and after IC from LA-NPC patients including 34 responders and 30 non-responders, as well as 50 plasma samples of healthy individuals, were tested using data-independent acquisition mass spectrometry. The proteins associated with clinical traits or IC benefits were investigated by weighted gene co-expression network analysis (WGCNA) and soft cluster analysis. Gene Ontology and Kyoto Encyclopedia of Genes and Genomes functional annotations were performed to determine the potential function of the identified proteins. The area under the receiver operating characteristic curve (AUC) was used to evaluate the performance of candidate biomarkers in predicting IC beneficiaries.

**Results:**

Compared with healthy individuals, 1027 differentially expressed proteins (DEPs) were found in the plasma of LA-NPC patients. Based on feedback from IC outcomes, 463 DEPs were identified in the pre-IC plasma between responders and non-responders. A total of 1212 DEPs represented the proteomic changes before and after IC in responders, while 276 DEPs were identified in post-IC plasma between responders and non-responders. WGCNA identified nine protein co-expression modules correlated with clinical traits. Soft cluster analysis identified four IC benefits-related protein clusters. Functional enrichment analysis showed that these proteins may play a role in IC *via* immunity, complement, coagulation, glycosaminoglycan and serine. Four proteins differentially expressed in all group comparisons, paraoxonase/arylesterase 1 (PON1), insulin-like growth factor-binding protein 3 (IGFBP-3), rheumatoid factor D5 light chain (v-kappa-3) and RNA helicase (DDX55), were associated with clinical traits or IC benefits. A four-protein model accurately identified potential IC beneficiaries (AUC=0.95) while diagnosing LA-NPC (AUC=0.92), and the prediction performance was verified using the models to confirm the effective IC (AUC=0.97) and evaluate IC outcome (AUC=0.94).

**Conclusion:**

The plasma protein profiles among IC responders and non-responders were different. PON1, IGFBP3, v-kappa-3 and DDX55 could serve as potential biomarkers for early identification of IC beneficiaries for individualised treatment of LA-NPC.

## Introduction

Nasopharyngeal carcinoma (NPC) is an aggressive type of head and neck malignant disease with a relatively high rate of morbidity and metastasis (1). In 2020, the World Cancer Report released by the International Agency for Research on Cancer (IARC) estimated 133,354 new cases of NPC and 80,008 NPC-related deaths worldwide (2). Within China, the corresponding numbers for such cases in 2020 were 62,444 and 34,810, accounting for 46.83% and 42.72% of the global total, respectively (3). Although progress in medical technology has made it possible to cure patients with early-stage NPC, more than 70% of patients were diagnosed as locoregionally advanced (LA)-NPC at initial presentation (4). Treatment fails for ~30–40% of LA-NPC patients, and tumour relapse or distant metastasis reduces the overall survival rate of these patients to 20% (5, 6). Therefore, improving the clinical treatment of LA-NPC is crucial for effectively controlling disease progression and reducing mortality.

In recent years, data from several large-scale clinical trials demonstrated that induction chemotherapy (IC) prior to radiotherapy of LA-NPC leads to improved survival outcomes (7–9). When employing IC as a National Comprehensive Cancer Network (NCCN) guideline recommendation for the treatment of LA-NPC, clinical analysis revealed that the short-term tumour response to IC varies significantly among individuals; symptoms of ~30% of LA-NPC patients receiving IC were not effectively alleviated, and IC only achieved 8% absolute survival benefit, indicating that numerous patients underwent ineffective treatment (8, 10, 11). Furthermore, the incidence of acute adverse grade 3 or 4 events triggered by IC was as high as 75% (7). Hematotoxicity, weight loss, oral mucositis and salivary gland dysfunction could postpone or interrupt follow-up treatment, thus increasing the risk of cancer progression and chemoresistance (12). Therefore, identifying in advance patients likely to have a favourable response to IC would be of great importance for individualised treatment of LA-NPC. Currently, clinical indicators cannot provide strategies for identifying patients suitable for IC before treatment, because existing evidence of Epstein-Barr virus (EBV) only supports its use in the diagnosis and prognosis of NPC, while the Response Evaluation Criteria in Solid Tumours (RECIST) value is limited to the determination of short-term efficacy after IC (13). Therefore, there is an urgent need to establish a biomarker-driven laboratory method for more precise IC application.

Proteins control all physiological processes including immunity, material exchange, metabolism and signal pathway regulation (14). During cancer progression, protein synthesis and the decomposition of tumour tissue are enhanced, which affects the expression and composition of circulating proteins. These abnormalities are accompanied by dynamic changes in tumour cell apoptosis or proliferation during treatment. Therefore, circulating proteins have been studied for their utility as biomarkers in a wide range of malignancies and disorders (15, 16). High-throughput technology has been employed to develop biomarkers for early diagnosis, chemo- and radio-resistance, and prognosis of NPC in the field of proteomics (17–19). However, the circulating protein profiles of LA-NPC patients with different IC efficacy, and the proteomic biomarkers for predicting the short-term efficacy of IC have not been reported.

In the present study, we investigated the plasma protein profiles of LA-NPC patients with different IC efficacy using data-independent acquisition (DIA) mass spectrometry, and conducted in-depth screening for differentially expressed proteins (DEPs), as well as the correlative analysis between pre-treatment plasma proteins, clinical traits and IC benefits. Then, we explored whether pre-treatment plasma proteins have the potential as biomarkers for early identification of IC beneficiaries.

## Material and Methods

### Participants, Samples and Grouping

We recruited 50 healthy controls (HC group) and 64 patients with non-metastatic stage III−IVa LA-NPC patients (LA-NPC group) at Yuebei People’s Hospital, Shantou University Medical College (Shaoguan, China), between May 2020 and January 2021. The detailed descriptions of participants are shown in **Table 1**. The distributions of age, gender, clinical stage, T- and N-classification, and EBV DNA load between patients showed no statistical differences. All patients were diagnosed under the guidance of the 8th edition of the American Joint Committee on Cancer (AJCC) Staging Manual (20). Patients participating in this study were not suffering from any other major diseases at the time of diagnosis, and all were initially diagnosed and did not receive any anti-tumour therapy before blood sampling. Histopathological diagnosis was non-keratinising undifferentiated carcinoma in all cases.

**Table 1:** Demographics and baseline characteristics of enrolled participants.

	HC	LA-NPC		*P* value
		EFF	NEFF	
**Population size**	50	34	30	
**Age**	50.18 ± 10.69	48.74 ± 11.35	51.30 ± 13.68	0.680
**Gender-no. (%)**				0.240
Male	37 (74)	22 (64.71)	25 (83.33)	
Female	13 (26)	12 (35.29)	5 (16.67)	
**Clinical stage-no. (%)**				0.518
III		20 (58.82)	20 (66.67)	
IVa		14 (41.18)	10 (33.33)	
**T Classification-no. (%)**				0.563
T1		0 (0)	1 (3.33)	
T2		10 (29.41)	9 (30.00)	
T3		11 (32.35)	12 (40.00)	
T4		13 (38.24)	8 (26.67)	
**N Classification-no. (%)**				0.289
N0		2 (5.88)	1 (3.33)	
N1		6 (17.65)	1 (3.33)	
N2		23 (67.65)	25 (83.33)	
N3		3 (8.82)	3 (10.00)	
**EBV DNA load (copies/mL)**				0.559
<1000		26 (76.47)	21 (70.00)	
>1000		8 (23.53)	9 (30.00)	

no. (%), number; EBV, Epstein-Barr virus.

To ensure that the identified proteins are relevant to IC efficacy, we only enrolled patients who received IC alone and who were administrated with the same regimen before radiotherapy. For IC, 75 mg/m^2^ docetaxel and 80 mg/m^2^ cisplatin (DP regimen) were given once every 3 weeks for two cycles. Prior to radiotherapy, we evaluated the tumour response to IC using RECIST (version 1.1) (21). Based on the measurement of gross tumour volume (GTV) using an Elekta Unity dedicated treatment planning system (TPS) and Monaco (v5.40.01) (22), we defined patients with complete and partial responses (CR/PR) as the effective (EFF) cohort, and patients with stable and progressive disease (SD/PD) as the non-effective (NEFF) cohort.

Plasma sampling was performed following the Human Proteome Organization (HUPO) recommendations (23). A total of 64 pairs of fasting blood samples were collected at two timepoints before and after IC. For plasma separation, peripheral blood was centrifuged under anticoagulant conditions at 1300 g for 10 min at 4°C, and the supernatant was transferred to an Eppendorf tube and stored at -80°C. Based on sampling times, we further divided the EFF cohort into pre-treatment effective (pre-EFF) and post-treatment effective (post-EFF) groups, and the NEFF cohort into pre-treatment non-effective (pre-NEFF) and post-treatment non-effective (post-NEFF) groups. This study was conducted in compliance with the ethical guidelines of the Helsinki Declaration, and was approved by the Institutional Review Board of Yuebei People’s Hospital, Shantou University Medical College (KY-2020-020). All participants gave written informed consent.

### Sample Preparation

Briefly, high abundance and low abundance proteins were separated from the plasma pools using a Multiple Affinity Removal LC Column-Human 14 (Hu14; Agilent Technologies), and their components were desalted and concentrated using a 5 kDa cut-off ultrafiltration device (Sartorius Corp). Samples were mixed with SDT buffer comprising 4% sodium dodecyl sulphate (SDS), 100 mM dithiothreitol (DTT) and 150 mM Tris-HCl pH 8.0, boiled for 15 min, centrifuged at 14,000 g for 20 min, and the supernatant was quantified using a BCA Protein Assay Kit (Bio-Rad Corp). Samples were stored at -80°C for subsequent experiments.

For digestion, 200 μg of proteins was repeatedly ultrafiltered with UA buffer (8 M Urea,150 mM Tris-HCl, pH 8.0) according to the filter-aided sample preparation (FASP) procedure to remove detergent and low-molecular-weight components. Next, 100 μL of 100 mM iodoacetamide (IAA) in UA buffer was added to block reduced cysteine residues and samples were incubated for 30 min in darkness. Filters were washed with 100 μL UA buffer three times then with 100 μL 25 mM NH_4_HCO_3_ buffer twice. The obtained protein suspensions were digested with 4 μg trypsin (Promega Corp) in 40 μL 25 mM NH_4_HCO_3_ buffer overnight at 37°C, and the resulting peptides were collected as a filtrate. The peptide content of each sample was estimated by UV light spectral density at 280 nm after desalting on an Empore SPE C18 cartridge (standard density, bed I.D. 7 mm, volume 3 mL; Sigma Corp), concentrated by vacuum centrifugation, and reconstituted in 40 µL of 0.1% (v/v) formic acid.

Digested pool peptides were subsequently fractionated using a High pH Reversed-phase Peptide Fractionation Kit (Thermo Scientific Corp) into 10 fractions. Each fraction was concentrated by vacuum centrifugation and reconstituted in 15 µL of 0.1% (v/v) formic acid. Collected peptides were desalted and reconstituted successively with a C18 cartridge and 40 µL of 0.1% (v/v) formic acid. Finally, iRT-Kits (Biognosys Corp) were added to correct the relative retention time differences between runs at a volume ratio of 1:3 for iRT standard peptides versus sample peptides.

### Data-Dependent Acquisition Mass Spectrometry

Peptide fractions for DDA library generation were analysed using a Q Exactive HF X mass spectrometer (Thermo Scientific Corp) coupled to an Easy nLC 1200 chromatography system (Thermo Scientific Corp). Peptide samples (1.5 μg) were first loaded onto an EASY-Spray C18 Trap column (P/N 164946, 3 μm, 75 μm × 2 cm; Thermo Scientific Corp), then separated on an EASY-Spray C18 LC Analytical Column (ES802, 2 μm, 75 μm × 25 cm; Thermo Scientific Corp) over a 120 min gradient from buffer B (0.1% formic acid and 84% acetonitrile). The column flow rate was maintained at 250 nL/min. MS detection was performed in positive ion mode, full scans were performed between 300 and 1800 m/z, the resolution for the MS1 scan was 60,000 at 200 m/z, the automatic gain control (AGC) target for the MS scan was set to 3e6, and the maximum injection time (IT) was 25 ms. The dynamic exclusion was set to 30.0s. Each full mass spectrometry (MS)-selected ion monitoring (SIM) scan followed 20 ddMS2 scans. The MS2 scan was performed at 15,000 resolution, the AGC target was 5e4, the maximum IT was 25 ms, and the collision energy was 30 eV.

### DIA Mass Spectrometry

Liquid chromatography-tandem mass spectrometry (LC-MS/MS) running in DIA mode (Shanghai Applied Protein Technology Co., Ltd) was used for analysis of sample peptides. Each DIA cycle was comprised of one full MS-SIM scan, and 30 DIA scans covered a mass range of 350−1,800 m/z. For full scans, the resolution was 120,000 at 200 m/z, the AGC target was 3e6, and the maximum IT was 50 ms. DIA scans were performed at 15,000 resolution, and the AGC target and maximum IT were set to 3e6 and auto, respectively. The collision energy was 30 eV and the runtime was 120 min, with a linear gradient of buffer B (84% acetonitrile and 0.1% formic acid) at a flow rate of 250 nL/min. MS performance monitoring was conducted by injection of QC samples (pooled samples from equal aliquots of each sample in the experiment) in DIA mode at the beginning of the MS study, and after every six injections throughout the experiment.

### Mass Spectrometry Data Analysis

To process and analyse the raw DDA data, Spectronaut 14.4.200727.47784 (Biognosys Corp) was used for FASTA sequence database searching, and the database was downloaded from the web at http://www.uniprot.org. The iRT peptide sequence was added to the database (>Biognosys|iRTKit|Sequence_fusionLGGNEQVTRYILAGVENSKGTFIIDPGGVIRGTFIIDPAAVIRGAGSSEPVTGLDAKTPVISGGPYEYRVEATFGVDESNAKTPVITGAPYEYRDGLDAASYYAPVRADVTPADFSEWSKLFLQFGAQGSPFLK). Regarding parameters, trypsin was used as the digestion enzyme, and max missed cleavages was set to 2. Carbamidomethyl (C) was specified as a fixed modification, and oxidation (M) and acetyl (protein N-term) were specified as dynamic modifications. All reported data were based on 99% confidence for protein identification as determined by false discovery rate [FDR = N(decoy)*2/(N(decoy) + N(target))] ≤1%. Finally, Spectronaut Pulsar X TM_12.0.20491.4 (Biognosys Corp) loaded with the original raw files and DDA searching results was used to construct the spectral library.

### Bioinformatics and Statistical Analyses

All statistical testing was performed using R version 4.1.1 (R Foundation for Statistical Computing) software packages and SPSS Statistics version 22.0 (IBM Corp.). To mine the proteomic differences between IC responders and non-responders, proteins with either increased or decreased relative abundance in the inter-group comparisons were defined as DEPs according to fold change ≥1.2 or ≤0.833 and p-value <0.05. A weighted protein co-expression network was constructed to identify hub modules related to clinical traits among the identified proteins using the Weighted Gene Co-Expression Network Analysis (WGCNA) package for R software. The fuzzy C-means algorithm, a software package termed Mfuzz (http://mfuzz.sysbiolab.eu/) based on the open-source statistical language R, was used for soft clustering analysis to observe changes in the expression of plasma proteins in the EFF cohort before and after IC. For DEPs and proteins related to clinical traits or IC benefits, Gene Ontology (GO) and Kyoto Encyclopedia of Genes and Genomes (KEGG) functional annotation were performed to explore the enriched functions and pathways using the clusterProfiler R package (24). Receiver operating characteristic (ROC) curve analysis was conducted to test the discriminative ability of the prediction models for IC beneficiaries.

Shapiro-Wilk normality tests were used to confirm whether data obeyed a normal distribution. For statistical comparison, one-way analysis of variance or Student t-tests were used for parameter data, chi-square tests were used for composition ratio, mean ± standard deviations were used to describe continuous variables, and *p <*0.05 for two-tailed tests was considered significant.

## Results

### IC Outcomes and Quality Control Data

**Figure 1:**
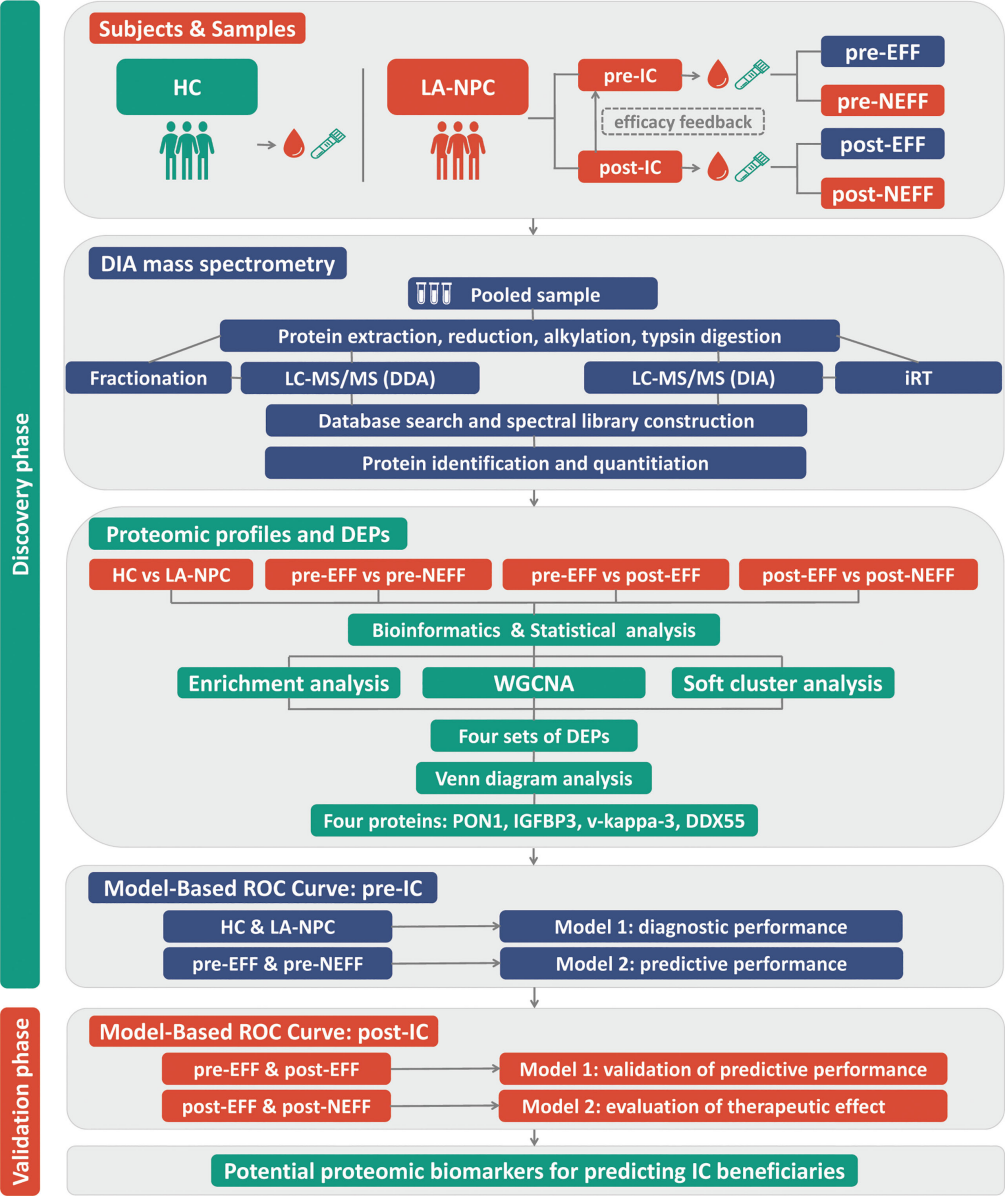
Roadmap for identifying biomarkers for early identification of IC beneficiaries. HC, healthy control; LA-NPC, locoregionally advanced nasopharyngeal carcinoma; IC, induction chemotherapy; pre-EFF, pre-effective; pre-NEFF, pre-non-effective; post-EFF, post-effective; post-NEFF, post-non-effective; DDA, data-dependent acquisition; DIA, data-independent acquisition; DEPs, differentially expressed proteins; WGCNA, weighted gene co-expression network analysis; ROC, receiver operating characteristic.

**Figure 2:**
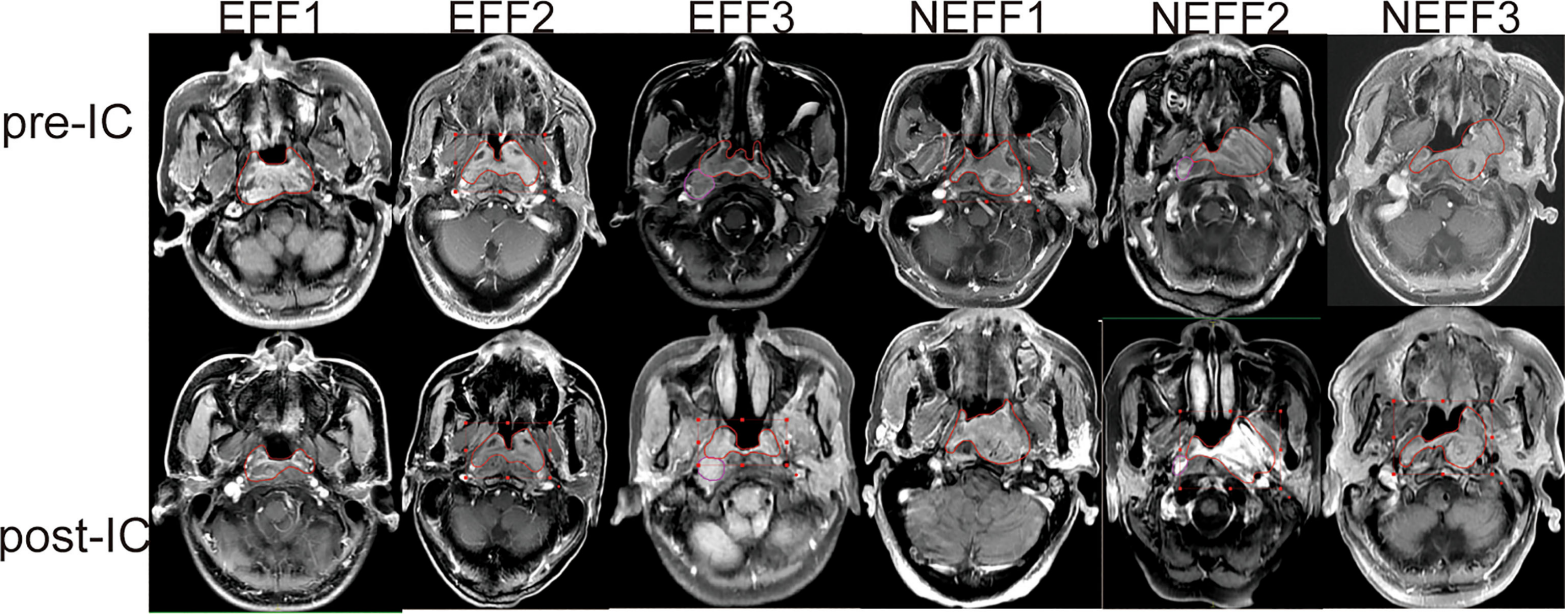
Typical MRI images for GTV changes before and after IC. MRI images of three patients with PR (EFF1-3), one patient with PD (NEFF1), and two patients with SD (NEFF2-3) at baseline (pre-IC) and after two cycles of IC (post-IC). The area within the red line is the primary focus. MRI, magnetic resonance imaging; GTV, gross tumour volume; IC, induction chemotherapy; EFF, effective; NEFF, non-effective.

The detailed roadmap for this study is shown in **Figure 1**. Based on the IC outcomes of the 64 enrolled patients, we classified one patient with CR and 33 patients with PR as the EFF cohort, and 29 patients with SD and 1 patient with PD as the NEFF cohort. **Figure 2** shows the typical changes in GTV before and after IC for 6 patients (PR = 3, PD = 1, SD = 2) assessed by magnetic resonance imaging, and GTV measurements for all patients are provided in **Supplementary Table 1**. Quality control data are shown in **Supplementary Figures 1A–D**. For protein identification, the FDR method was applied for multiple testing correction, and the quantitative results with a Q value <0.01 indicate high reliability. The intensity jitter distribution plot includes a few outlier samples, and the strength of the correlation between quality control samples was greater than 0.9 in all cases. A total of 2512 proteins were identified across all groups, indicating high repeatability and quantitative stability.

### Differences in Protein Profiles Between IC Responders and Non-Responders

**Figure 3:**
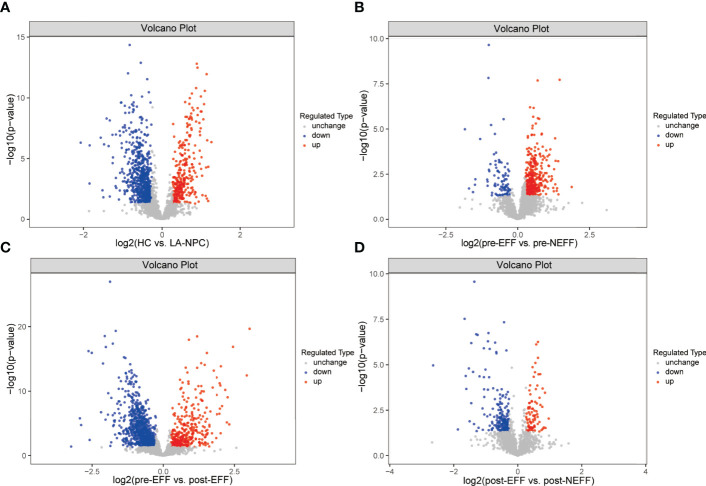
Volcano map of differentially expressed proteins (DEPs). Red represents upregulated DEPs and blue represents downregulated DEPs. **(A)** DEPs between HC and LA-NPC groups. **(B)** DEPs between pre-EFF and pre-NEFF groups. **(C)** DEPs between pre-EFF and post-EFF groups. **(D)** DEPs between post-EFF and post-NEFF groups.

To comprehensively understand the differences and changes of plasma protein profiles between responders and non-responders before and after IC, screening of DEPs was carried out in tiers. A total of 1027 proteins were found to be differentially expressed in the LA-NPC group compared with the HC group, of which 324 were upregulated and 703 were downregulated (**Figure 3A**). Subsequently, 463 DEPs, including 370 upregulated and 93 downregulated, were identified between the pre-EFF group and the pre-NEFF group (**Figure 3B**). **Figure 3C** shows 1212 DEPs (380 upregulated and 832 downregulated) identified between pre-EFF and post-EFF groups, while **Figure 3D** shows 276 DEPs (86 upregulated and 190 downregulated) between post-EFF and post-NEFF groups.

**Figure 4:**
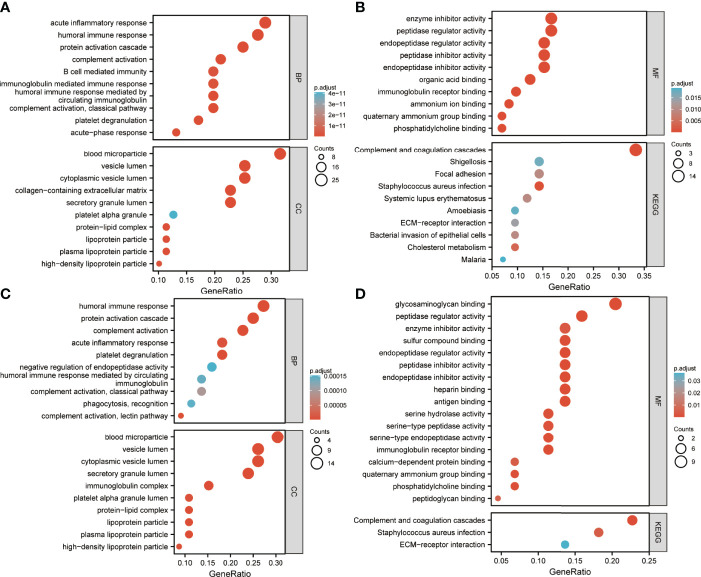
Gene Ontology (GO) and Kyoto Encyclopedia of Genes and Genomes (KEGG) functional enrichment analyses of HC vs. LA-NPC and pre-EFF vs. pre-NEFF DEPs. The ordinates in A−D represent enriched GO functional classification terms, including biological process (BP), cellular component (CC) and molecular function (MF) categories, and KEGG pathways. The abscissa represents terms enriched for DEPs under each functional classification. The size of the bubble represents the number of DEPs, and its colour indicates the significance of the enriched terms based on Fisher’s accuracy. The *p*-value was calculated by Fisher’s Exact test, and the colour gradient represents the magnitude of the *p*-value, from cyan (large) to red (small). **(A, B)** Functional enrichment analysis results for DEPs between HC and LA-NPC groups. **(C, D)** Functional enrichment analysis results for DEPs between pre-EFF and pre-NEFF groups.

**Figure 5:**
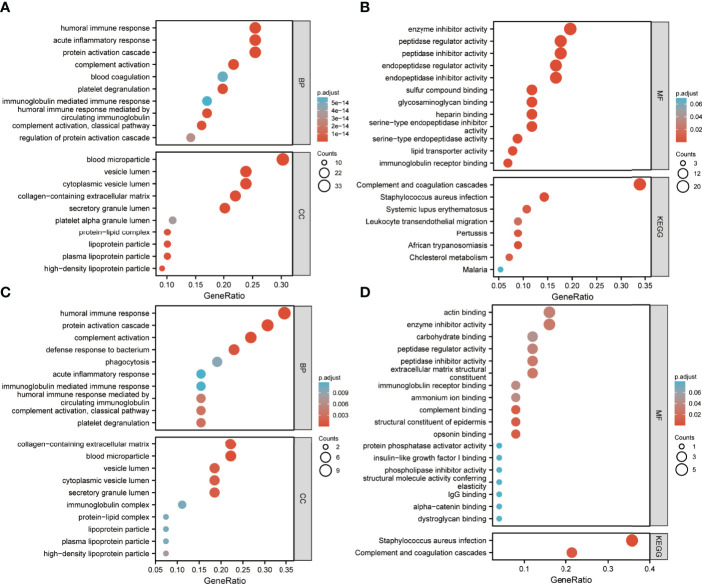
GO and KEGG functional enrichment analyses of pre-EFF vs. post-EFF and post-EFF vs. post-NEFF DEPs. The ordinates in **(A−D)** represent enriched GO functional classification terms, including biological process (BP), cellular component (CC) and molecular function (MF) categories, and KEGG pathway. The abscissa represents terms enriched for DEPs under each functional classification. The size of the bubble represents the number of DEPs, and its colour indicates the significance of the enriched terms based on Fisher’s accuracy. The *p*-value was calculated by Fisher’s Exact test, and the colour gradient represents the size of the *p*-value from cyan (large) to red (small). **(A, B)** Functional enrichment analysis results for DEPs between pre-EFF and post-EFF groups. **(C, D)** Functional enrichment analysis results for DEPs between post-EFF and post-NEFF groups.

Enrichment analysis of DEPs is shown in **Figures 4**, **5**. GO annotation showed that the highly enriched terms of biological process (BP), cellular component (CC), and molecular function (MF) categories in all batch data are roughly the same, while some MF terms, such as glycosaminoglycan binding, sulfur compound binding, heparin binding, serine-related enzyme activities, complement binding, structural constituents of the epidermis, and opsonin binding were enriched between pre-EFF and pre-NEFF groups, pre-EFF and post-EFF groups, and post-EFF and post-NEFF groups, respectively. In addition, KEGG analysis indicated that the complement and coagulation cascade was the most significantly enriched pathway for all batch data.

### Correlation Between Pre-Treatment Plasma Proteins and Clinical Traits

**Figure 6:**
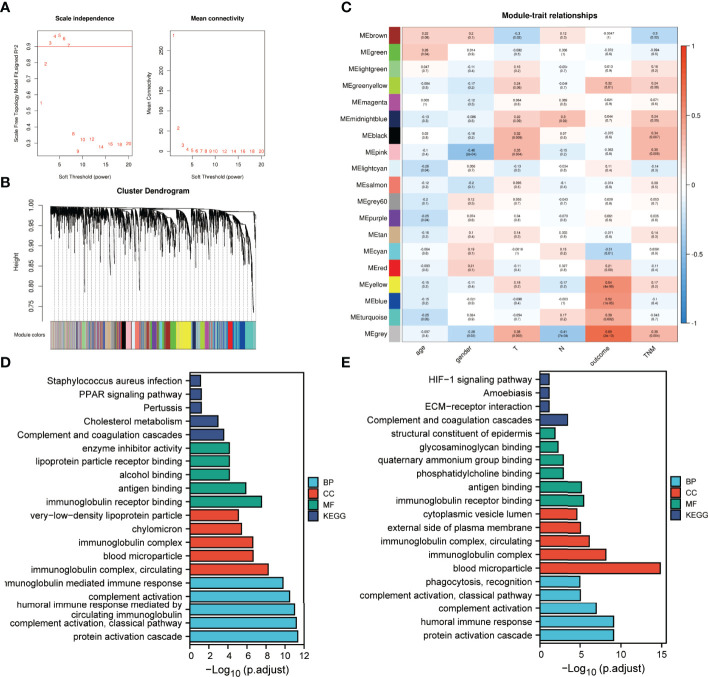
Protein modules associated with the clinical traits of LA-NPC identified by weighted gene co-expression network analysis (WGCNA). **(A)** Analysis of scale-free index and mean connectivity for various soft-threshold powers. **(B)** Dendrogram of all identified proteins clustered based on dissimilarity through consensus topological overlap with the corresponding module. Coloured rows correspond to the 19 modules identified. **(C)** Heatmap of correlations between module eigen proteins and clinical traits of LA-NPC. **(D)** Functional enrichment analysis of proteins in modules associated with tumour stage. **(E)** Functional enrichment analysis of proteins in modules associated with IC outcome.

To investigate correlations between the identified proteins and clinical traits, a weighted protein co-expression network was constructed from 2297 proteins after filtering out proteins expressed in fewer than half of the samples. The power of β = 5 was selected for soft thresholding to ensure a scale-free topology (**Figure 6A**). Among the 19 co-expression modules, proteins contained in the grey module were not attributed to any modules (**Figure 6B**). Clinical traits, such as patient’s age, gender, T classification, N classification, clinical stage and IC outcome were used for correlation analysis. The results presented in **Figure 6C** indicate that the pink module was negatively correlated with gender. The modules negatively correlated with T classification and clinical stage are brown, while the positive correlations are coloured black and pink. The midlightblue module represents a positively correlation with N classification. Only the cyan module is negatively correlated with IC outcome, while greenyellow, yellow, blue and turquoise modules are positively correlated with IC outcome.

Modules significantly correlated with tumour stage and IC outcome were integrated separately using the criteria correlation >0.3 and *p* <0.001 to perform enrichment analysis (**Figures 6D, E**). GO annotation showed that the overrepresented BP, CC and MF terms for the two batches were roughly the same, and similar to the annotation results for DEPs. KEGG analysis indicated that the complement and coagulation cascade was still the most significantly enriched pathway for the two batches of data.

### Correlation Between Pre-Treatment Plasma Proteins and IC Benefit

**Figure 7:**
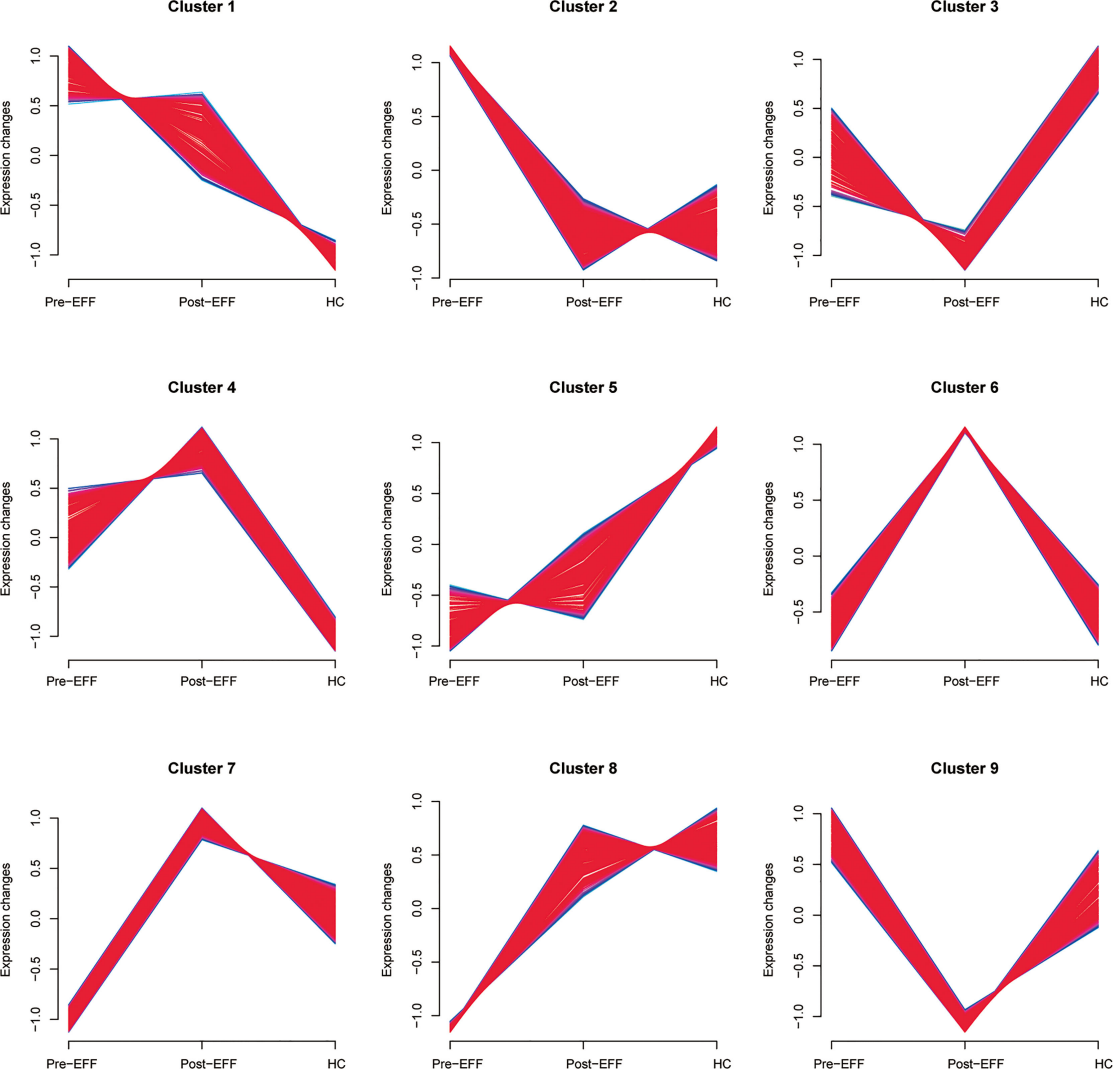
Nine protein clusters correlated with IC benefits identified by soft cluster analysis. The horizontal axis represents groups and the vertical axis represents expression changes in proteins around the time of IC in the EFF cohort. Protein expression changes in cluster 1 and cluster 2 show a high positive correlation with IC benefits, and protein expression changes in cluster 7 and cluster 8 showed a low positive correlation with IC benefits.

To determine whether the identified proteins are correlated with IC benefits, nine clusters that tended to exhibit significant changes were identified by soft clustering analysis to reveal proteomic changes in the EFF cohort around the time of IC (**Figure 7**). In cluster 1, expression of proteins in the pre-EFF group was significantly higher than that in the HC group, and it was decreased significantly in the post-EFF group. Similarly, proteins in cluster 2 were also highly expressed in the pre-EFF group compared with the HC group, but tended to be normal in the post-EFF group. By contrast, protein expression levels in clusters 7 and 8 in the pre-EFF group were significantly lower than that in the HC group, but in the post-EFF group, protein expression levels for cluster 7 were significantly higher than that in the HC group, while protein expression levels for cluster 8 tended to be normal. These expression changes appear to be associated with tumour regression (i.e. PR and CR, respectively).

For the other clusters, the changes in proteins expression after IC in clusters 3, 4 and 6 did not show a strong correlation with the benefits of IC. Although the changes in proteins expression in cluster 5 showed a similar trend to clusters 7 and 8, a considerable proportion of proteins could be found whose expression did not change significantly after IC. In addition, the protein expression in the pre-EFF group in cluster 9 did not show significant differences compared to the HC group, and their expression in the post-EFF group was significantly lower than that in the HC group. Therefore, we did not include the proteins in these clusters in further analyses.

**Figure 8:**
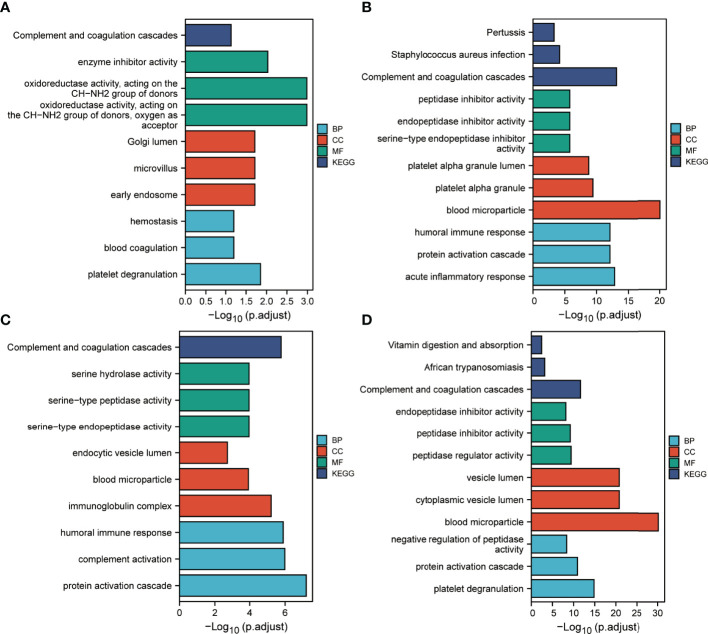
Functional enrichment analysis of IC benefits-related proteins. **(A–D)** Results corresponding to functional enrichment analysis of proteins in clusters 1, 2, 7 and 8, respectively.

We performed enrichment analysis of proteins contained in clusters 1, 2, 7 and 8 (**Figure 8**). GO annotation showed that the most significantly enriched terms were similar to those identified in the DEPs annotation results. KEGG analysis indicated that the complement and coagulation cascade was the most enriched pathway.

#### Identification of Predictive Biomarkers for IC Beneficiaries

**Figure 9:**
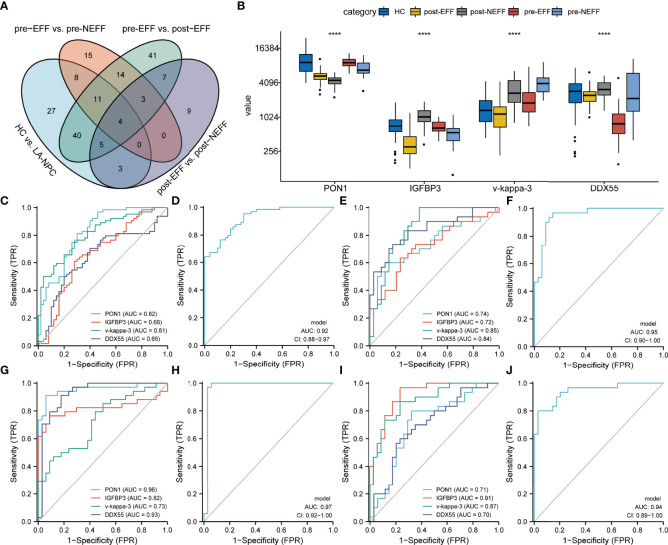
Identification of candidate biomarkers. **(A)** Venn diagram showing the overlap of DEPs between the four compared datasets. **(B)** Relative protein abundances of PON1, IGFBP3, v-kappa-3 and DDX55. **(C, D)** ROC analysis of the four proteins and their co-constructed model for distinguishing between HC and LA-NPC groups. **(E, F)** ROC analysis of the four proteins and their co-constructed model for distinguishing between pre-EFF and pre-NEFF groups. **(G, H)** ROC analysis of the four proteins and their co-constructed model for distinguishing between pre-EFF and post-NEFF groups. **(I, J)** ROC analysis of the four proteins and their co-constructed model for distinguishing between post-EFF and post-NEFF groups. ****p < 0.0001

DEPs with a given gene name in all datasets overlapped, and four proteins were identified (**Figure 9A**); paraoxonase/arylesterase 1 (PON1), insulin-like growth factor-binding protein 3 (IGFBP3), rheumatoid factor D5 light chain (v-kappa-3), and RNA helicase (DDX55). Their relative protein abundances are shown in **Figure 9B**. Based on the results of the above analyses, the brown module to which PON1 belongs was negatively correlated with clinical stage and T classification (**Figure 6C**). For v-kappa-3, the turquoise module in which it is located was correlated with IC outcome, and its expression change in the EFF cohort around the time of IC is correlated with IC benefits, as observed for cluster 2 (**Figures 6C**, **7**). The blue module containing DDX55 was also correlated with IC outcome (**Figure 3C**). The expression changes for DDX55 in the EFF cohort around the time of IC presented a positive correlation with IC benefits, as shown for cluster 8 (**Figure 7**).

To explore the potential of the four proteins as biomarkers, we performed multivariate logistic regression analysis and ROC curve analysis. When applying the four proteins to differentiate HC and LA-NPC groups, the AUC values for PON1, IGFBP3, v-kappa-3 and DDX55 were 0.82, 0.66, 0.81 and 0.65, respectively (**Figure 9C**). When the four proteins were combined into a diagnostic model, the AUC value was 0.92, higher than for single protein models (**Figure 9D**). The corresponding AUC values of the four proteins for distinguishing between pre-EFF and pre-NEFF groups were 0.74, 0.72, 0.85 and 0.84, while the AUC value of the efficacy prediction model composed of these four proteins was increased to 0.95 (**Figures 9E, F**).

To further verify the accuracy of these proteins for identifying IC beneficiaries before treatment, we also tested their ability to confirm the effective IC and evaluate IC outcome. **Figure 9G** shows that the AUC values of the four proteins for discriminating between pre-EFF and post-EFF groups were 0.96, 0.82, 0.73 and 0.93, respectively. Compared with single protein models, the AUC value of the model integrating all four proteins was 0.97 (**Figure 9H**). Furthermore, the results presented in **Figures 9I, J** show that the four proteins could readily discriminate between post-EFF and post-NEFF groups (PON1, AUC = 0.71; IGFBP3, AUC = 0.91; v-kappa-3, AUC = 0.87; DDX55, AUC = 0.70), especially the model combining all four proteins (AUC = 0.94).

## Discussion

For LA-NPC, the main advantages of IC are eliminating micro-metastases and shrinking primary tumours to improve the outcome of follow-up radiotherapy (7–10). However, despite this treatment, the short-term tumour response rate and long-term survival rate are not improved for some patients, and serious systemic toxicity can occur following IC (11, 12). Therefore, early identification of patients suitable for IC before treatment is the ideal strategy to improve the effective rate of clinical treatment of LA-NPC. In other words, patients who do not respond to one IC regimen could be rapidly subjected to other regimens. Currently, no clear omics data were available on the molecular basis of IC-sensitivity and -resistance for LA-NPC. To address this issue, we comprehensively evaluated the plasma protein profiles of responders and non-responders around the time of IC using a DIA approach, and identified IC benefit-related proteins. To the best of our knowledge, this is the first study to apply a high-throughput proteomics approach to investigate the blood molecular characteristics of LA-NPC patients with different IC efficacy.

In order to mining the potential correlation between plasma proteins and the short-term efficacy of IC as much as possible, we explored various elements. IC comprises combinations of different drugs, usually from two or more drug classes. Considering that existing chemotherapeutics work through various mechanisms and sites of action, we unified the medication regimen and treatment cycle to avoid differences in efficacy caused by different regimens or dose intensities (25). Moreover, previous studies on biomarkers predicting the efficacy of IC included patients receiving concurrent chemoradiotherapy, while radiotherapy was proved to cover the efficacy of IC (13, 19, 26). Therefore, we only enrolled patients who received IC alone prior to radiotherapy in the present study, and focused on the short-term efficacy of IC by sampling before radiotherapy. Eventually, compared with other studies using only pre-treatment samples to identify predictive biomarkers of chemotherapy efficacy, we also utilised matched post-IC samples to confirm the correlation between pre-treatment plasma proteins and IC benefits, and to verify the potential of pre-treatment plasma proteins to develop efficacy predictive biomarkers (13, 15, 19).

Based on this design, we observed different protein profiles in the pre-treatment plasma between IC responders and non-responders, and the protein profiles of the two remained differences after changes were produced under the intervention of IC. In addition, a number of proteins were found to be related to clinical traits or IC benefits. In the results of enrichment analysis, GO annotation revealed that many functional terms were repeatedly highlighted all batch data. Thus, we infer those proteins enriched in these terms may jointly participate in the occurrence and development of NPC. However, in terms of immune response, the results indicate that chemotherapy drugs may not only inhibit tumour cells and activate immune effectors, but also promote the release of some inflammatory and angiogenesis-related cytokines, which may explain why chemotherapy has no effect on some tumours (27). It is notable that the location of some functional terms in the data may imply their potential relevance to IC benefits. For example, glycosaminoglycan assembly in the Golgi is sensitive to drug treatment and a variety of environmental factors, which can alter the structure of glycosaminoglycans and thereby promote or inhibit different signalling pathways (28). In addition, serine synthesis can help hypoxic cancer stem cells to regulate redox state (29). Serine restriction and inhibiting its signalling pathway can reduce the proliferative activity of cancer cells (30). KEGG analysis revealed that complement activation and coagulation functions can enhance the malignant biological behaviours of cancer, such as inflammation, epithelial-mesenchymal transition (EMT) and angiogenesis (31). Some genes in the complement and coagulation cascade signalling pathway are considered predictors of chemosensitivity (32).

The above analysis indicates that LA-NPC circulating proteins are involved in a series of complex mechanisms in the process of IC, including protein-protein interactions and drug–protein target interactions. Our results lay a foundation for further revealing the precise molecular mechanism and new therapeutic targets. The differences in plasma protein profiles between responders and non-responders after IC reflect the different sensitivity of various subpopulations of NPC cells to chemotherapeutic drugs caused by tumor heterogeneity (33). Based on the efficacy feedback, we confirmed the potential correlation between pre-treatment plasma proteins and short-term efficacy of IC by screening of DEPs and bioinformatics analysis. Although some of the proteins we identified may not be NPC-specific, their expression may be regulated by pro- or anti-oncogenes, as well as other tumour-associated genes and their products, which can indirectly reflect the state of the tumor (34).

Considering the above-mentioned problems of IC for LA-NPC, we further confirmed the potential clinical value of pre-treatment plasma proteins by identifying efficacy predictive biomarkers. The four candidate biomarkers identified in this study have been linked to physiological processes or chemotherapy resistance related to malignant diseases (35–43). PON1 is a calcium-dependent hydrolase protein mainly involved in the occurrence and development of cancers through the regulation of oxidative stress and inflammation (35). PON1 is expressed at low levels in the blood of patients with a variety of cancers, and levels are further decreased after treatment (35, 36). We observed similar trends in the present study. The low expression of PON1 in LA-NPC plasma may be caused by inhibition of its synthesis in liver and/or oxidative stress, and the continuous decrease in levels after IC may be caused by inhibition of PON1 synthesis in the liver by chemotherapeutic drugs inhibiting peroxisome proliferator-activated receptor delta (37). A previous study found that decreased serum PON1 was significantly correlated with tumour load, consistent with our findings that PON1 levels were negatively correlated with clinical stage and T classification (35). Enrichment analysis showed that PON1 was enriched in terms related to lipids and cholesterol, suggesting that preventing low-density lipoprotein oxidation, maintaining cholesterol homeostasis, and reducing chronic inflammation may be effective ways to prevent the further deterioration of LA-NPC.

IGFBP3 is an N-linked glycosylated, phosphorylated, secretory protein correlated with multiple cancers. In this study, we found that the expression of IGFBP3 in LA-NPC was lower than in the HC group, and even lower in the pre-NEFF group. It is interesting to note that IGFBP3 was further downregulated in the post-EFF group, but significantly upregulated in the post-NEFF group and higher than that in the HC group. The previous studies found that upregulation of IGFBP3 promotes the proliferation, invasion and metastasis of NPC cells, consistent with our finding that IC non-responders may have a poor prognosis (38, 39). Enrichment analysis found that IGFBP3 may play a role in IC through binding insulin-like growth factor I (IGF-1). IGF-1 can promote progress of the cell cycle, leading to mitosis and anti-apoptosis, and phosphorylation of IGFBP3 can enhance its binding to IGF-1 (40). In addition, IGFBP3 can enhance TGF-β1-mediated EMT by activating other cells against decapentaplegic homolog 2/3 (SMAD2/3) (38). EMT is known to involve both cisplatin and docetaxel resistance mechanisms, which further indicates the potential of IGFBP3 as an efficacy predictive biomarker (41).

Compared with the above two proteins, the reports of v-kappa-3 in cancer are very limited, which may be related to its unknown biological function. DDX55 is a member of the RNA helicase family, members of which perform crucial roles in cell metabolism. Abnormally high expression of DDX55 can be used as a reference indicator for poor prognosis of a variety of cancers (42, 43). Similar to these studies, we also found that the expression of DDX55 in the pre-NEFF group was higher than that in the HC group. In lung cancer, DDX55-associated genes were found to be enriched in proto-oncogene (MYC) targets, and MYC transcription can be regulated by cytokines (42). Considering that NPC cells can also stimulate the secretion of cytokines, hence we speculated that differences in the expression of DDX55 in plasma from patients with differences in IC efficacy is related to the modulation of RNA binding and ATP-dependent helicase activity by small molecules, or disrupting certain protein-protein interactions implicated in cancer progression (43).

In the performance evaluation of candidate biomarkers, we found that the model composed of all four proteins showed satisfactory accuracy in diagnosing LA-NPC, predicting IC efficacy, confirming effective IC, and evaluating IC outcome (all AUC values ≥0.92). It is worth noting that continuous and dynamic expression detection is one of the necessary conditions to determine whether biomarkers have the potential to predict efficacy, we used post-IC samples to validate the efficacy predictive biomarkers identified in pre-IC samples (44). The advantage of this is that it can confirm the post-treatment detectability and IC benefit relevance of candidate biomarkers in a way that the use of additional pre-IC samples alone does not (45). In addition, diagnostic model was constructed with the aim of detecting LA-NPC correlation of biomarkers. Predictive model was primarily used to assess the precision of biomarkers for identifying potential IC beneficiaries. The two models constructed using post-IC samples were used to confirm whether the potential IC beneficiaries identified by the biomarkers were indeed treated effectively, and to further exclude false positive results. Thus, the cohort design containing six groups, the four-tier in-depth screening of DEPs and multi model evaluation increased the reliability of our results and demonstrated the potential clinical value of the candidate biomarkers.

Although this study revealed differences in circulating protein profiles among LA-NPC patients with different IC outcomes, the identified potential biomarkers need to be further verified by more independent clinical cohorts due to the limitations of single-center nature. Furthermore, in addition to methods such as enzyme-linked immunosorbent assays, considering that IC usually involves two or more drugs which are administered to the patients at the same time, functional study of the potential biomarkers also needs to explore a reliable method to establish multidrug resistant cell line models.

## Conclusions

In summary, the present study comprehensively investigated the plasma protein profiles of LA-NPC patients with different IC outcomes and demonstrated the potential correlation between pre-treatment plasma proteins and the short-term efficacy of IC. The identified four proteins, PON1, IGFBP3, v-kappa-3 and DDX55, can achieve the early identification of IC beneficiaries while diagnosing LA-NPC, and they could confirm whether the identified potential beneficiaries have been effectively treated after IC, and distinguish responders and non-responders. These findings may help to reveal new targets of IC and serve the individualised treatment of LA-NPC. Whether the identified potential biomarkers are specific to the DP regimen remains to be clarified.

## Data Availability Statement

The original contributions presented in the study are included in the article/**Supplementary Material**. Further inquiries can be directed to the corresponding author.

## Ethics Statement

The studies involving human participants were reviewed and approved by The Institutional Review Board of Yuebei People’s Hospital, Shantou University Medical College (KY-2020-020). The patients/participants provided their written informed consent to participate in this study.

## Author Contributions

Conceptualization, J-CL, S-QZ and S-MP. Methodology, S-QZ. Software, JL. Formal analysis, all authors. Investigation, all authors. Writing—original draft preparation, S-QZ. Writing—review and editing, J-CL. Supervision, H-BC. Project administration, J-CL. All authors contributed to the article and approved the submitted version.

## Funding

This study was supported by grants from the Shaoguan Science and Technology Plan Projects in 2020, China (Grant no. 200812094530421).

## Conflict of Interest

The authors declare that the research was conducted in the absence of any commercial or financial relationships that could be construed as a potential conflict of interest.

## Publisher’s Note

All claims expressed in this article are solely those of the authors and do not necessarily represent those of their affiliated organizations, or those of the publisher, the editors and the reviewers. Any product that may be evaluated in this article, or claim that may be made by its manufacturer, is not guaranteed or endorsed by the publisher.

## References

[1] Chen YP Chan ATC Le QT Blanchard P Sun Y Ma J. Nasopharyngeal Carcinoma. Lancet (2019) 394(10192):64–80. doi: 10.1016/S0140-6736(19)30956-0 31178151

[2] Sung H Ferlay J Siegel RL Laversanne M Soerjomataram I Jemal A. Global Cancer Statistics 2020: GLOBOCAN Estimates of Incidence and Mortality Worldwide for 36 Cancers in 185 Countries. CA Cancer J Clin (2021) 71(3):209–49. doi: 10.3322/caac.21660 33538338

[3] Cancer Today. Population Fact Sheets (2020). Available at: https://gco.iarc.fr/today/fact-sheets-populations (Accessed December 10, 2021).

[4] Liu SL Bian LJ Liu ZX Chen QY Sun XS Sun R. Development and Validation of the Immune Signature to Predict Distant Metastasis in Patients With Nasopharyngeal Carcinoma. J Immunother Cancer (2020) 8(1):e000205. doi: 10.1136/jitc-2019-000205 32303611 PMC7204817

[5] Zhang W Guo Q Liu G Zheng F Chen J Huang D. NKILA Represses Nasopharyngeal Carcinoma Carcinogenesis and Metastasis by NF-κb Pathway Inhibition. PLoS Genet (2019) 15(8):e1008325. doi: 10.1371/journal.pgen.1008325 31430288 PMC6716677

[6] Lu S Yu Z Xiao Z Zhang Y. Gene Signatures and Prognostic Values of M^6^a Genes in Nasopharyngeal Carcinoma. Front Oncol (2020) 10:875. doi: 10.3389/fonc.2020.00875 32596151 PMC7300221

[7] Zhang Y Chen L Hu GQ Zhang N Zhu XD Yang KY. Gemcitabine and Cisplatin Induction Chemotherapy in Nasopharyngeal Carcinoma. N Engl J Med (2019) 381(12):1124–35. doi: 10.1056/NEJMoa1905287 31150573

[8] Sun Y Li WF Chen NY Zhang N Hu GQ Xie FY. Induction Chemotherapy Plus Concurrent Chemoradiotherapy Versus Concurrent Chemoradiotherapy Alone in Locoregionally Advanced Nasopharyngeal Carcinoma: A Phase 3, Multicentre, Randomised Controlled Trial. Lancet Oncol (2016) 17(11):1509–20. doi: 10.1016/S1470-2045(16)30410-7 27686945

[9] Cao SM Yang Q Guo L Mai HQ Mo HY Cao KJ. Neoadjuvant Chemotherapy Followed by Concurrent Chemoradiotherapy Versus Concurrent Chemoradiotherapy Alone in Locoregionally Advanced Nasopharyngeal Carcinoma: A Phase III Multicentre Randomised Controlled Trial. Eur J Cancer (2017) 75:14–23. doi: 10.1016/j.ejca.2016.12.039 28214653

[10] Liu LT Tang LQ Chen QY Zhang L Guo SS Guo L. The Prognostic Value of Plasma Epstein-Barr Viral DNA and Tumor Response to Neoadjuvant Chemotherapy in Advanced-Stage Nasopharyngeal Carcinoma. Int J Radiat Oncol Biol Phys (2015) 93(4):862–69. doi: 10.1016/j.ijrobp.2015.08.003 26530755

[11] Sun XS Xiao BB Lu ZJ Liu SL Chen QY Yuan L. Stratification of Candidates for Induction Chemotherapy in Stage III-IV Nasopharyngeal Carcinoma: A Large Cohort Study Based on a Comprehensive Prognostic Model. Front Oncol (2020) 10:255. doi: 10.3389/fonc.2020.00255 32185130 PMC7059214

[12] Peng H Chen B He S Tian L Huang Y. Efficacy and Toxicity of Three Induction Chemotherapy Regimens in Locoregionally Advanced Nasopharyngeal Carcinoma: Outcomes of 10-Year Follow-Up. Front Oncol (2021) 11:765378. doi: 10.3389/fonc.2021.765378 34722320 PMC8551638

[13] Lei Y Li YQ Jiang W Hong XH Ge WX Zhang Y. A Gene-Expression Predictor for Efficacy of Induction Chemotherapy in Locoregionally Advanced Nasopharyngeal Carcinoma. J Natl Cancer Inst (2021) 113(4):471–80. doi: 10.1093/jnci/djaa100 PMC8023816 33094348

[14] Altelaar AF Munoz J Heck AJ. Next-Generation Proteomics: Towards an Integrative View of Proteome Dynamics. Nat Rev Genet (2013) 14(1):35–48. doi: 10.1038/nrg3356 23207911

[15] Kubota D Mukaihara K Yoshida A Tsuda H Kawai A Kondo T. Proteomics Study of Open Biopsy Samples Identifies Peroxiredoxin 2 as a Predictive Biomarker of Response to Induction Chemotherapy in Osteosarcoma. J Proteomics (2013) 91:393–404. doi: 10.1016/j.jprot.2013.07.022 23911960

[16] Zhang L Wang W Zhang S Wang Y Guo W Liu Y. Identification of Lysine Acetylome in Cervical Cancer by Label-Free Quantitative Proteomics. Cancer Cell Int (2020) 20:182. doi: 10.1186/s12935-020-01266-z 32489318 PMC7247262

[17] Xiao L Xiao T Wang ZM Cho WC Xiao ZQ. Biomarker Discovery of Nasopharyngeal Carcinoma by Proteomics. Expert Rev Proteomics (2014) 11(2):215–25. doi: 10.1586/14789450.2014.897613 24611579

[18] Zhang G Zhang K Li C Li Y Li Z Li N. Serum Proteomics Identify Potential Biomarkers for Nasopharyngeal Carcinoma Sensitivity to Radiotherapy. Biosci Rep (2019) 39(5):BSR20190027. doi: 10.1042/BSR20190027 31040200 PMC6522734

[19] Liang Y Li J Li Q Tang L Chen L Mao Y. Plasma Protein-Based Signature Predicts Distant Metastasis and Induction Chemotherapy Benefit in Nasopharyngeal Carcinoma. Theranostics (2020) 10(21):9767–78. doi: 10.7150/thno.47882 PMC7449924 32863958

[20] Lydiatt WM Patel SG O'Sullivan B Brandwein MS Ridge JA Migliacci JC. Head and Neck Cancers-Major Changes in the American Joint Committee on Cancer Eighth Edition Cancer Staging Manual. CA Cancer J Clin (2017) 67(2):122–37. doi: 10.3322/caac.21389 28128848

[21] Eisenhauer EA Therasse P Bogaerts J Schwartz LH Sargent D Ford R. New Response Evaluation Criteria in Solid Tumours: Revised RECIST Guideline (Version 1.1). Eur J Cancer (2009) 45(2):228–47. doi: 10.1016/j.ejca.2008.10.026 19097774

[22] Ding S Li Y Liu H Li R Wang B Zhang J. Comparison of Intensity Modulated Radiotherapy Treatment Plans Between 1.5t MR-Linac and Conventional Linac. Technol Cancer Res Treat (2021) 20:1533033820985871. doi: 10.1177/1533033820985871 33472549 PMC7829462

[23] Tuck MK Chan DW Chia D Godwin AK Grizzle WE Krueger KE. Standard Operating Procedures for Serum and Plasma Collection: Early Detection Research Network Consensus Statement Standard Operating Procedure Integration Working Group. J Proteome Res (2009) 8(1):113–17. doi: 10.1021/pr800545q PMC2655764 19072545

[24] Wu T Hu E Xu S Chen M Guo P Dai Z. Clusterprofiler 4.0: A Universal Enrichment Tool for Interpreting Omics Data. Innovation (N Y) (2021) 2(3):100141. doi: 10.1016/j.xinn.2021.100141 PMC8454663 34557778

[25] Mitchell MJ Billingsley MM Haley RM Wechsler ME Peppas NA Langer R. Engineering Precision Nanoparticles for Drug Delivery. Nat Rev Drug Discov (2021) 20(2):101–24. doi: 10.1038/s41573-020-0090-8 PMC7717100 33277608

[26] Sun Y Guo W Bai Y Ge M Hu C Wu S. Neoadjuvant Dose-Modified Docetaxel in Squamous Cell Carcinoma of the Head and Neck: A Phase 3 Study. Oral Dis (2020) 26(2):285–94. doi: 10.1111/odi.13252 31830347

[27] Ghiringhelli F Apetoh L. The Interplay Between the Immune System and Chemotherapy: Emerging Methods for Optimizing Therapy. Expert Rev Clin Immunol (2014) 10(1):19–30. doi: 10.1586/1744666X.2014.865520 24308838

[28] Lan Y Li X Liu Y He Y Hao C Wang H. Pingyangmycin Inhibits Glycosaminoglycan Sulphation in Both Cancer Cells and Tumour Tissues. J Cell Mol Med (2020) 24(6):3419–30. doi: 10.1111/jcmm.15017 PMC7131950 32068946

[29] Samanta D Semenza GL. Serine Synthesis Helps Hypoxic Cancer Stem Cells Regulate Redox. Cancer Res (2016) 76(22):6458–62. doi: 10.1158/0008-5472 27811150

[30] Muthusamy T Cordes T Handzlik MK You L Lim EW Gengatharan J. Serine Restriction Alters Sphingolipid Diversity to Constrain Tumour Growth. Nature (2020) 586(7831):790–95. doi: 10.1038/s41586-020-2609-x PMC7606299 32788725

[31] Conway EM. Reincarnation of Ancient Links Between Coagulation and Complement. J Thromb Haemost (2015) 13(Suppl 1):S121–32. doi: 10.1111/jth.12950 26149013

[32] Zhang J Chen M Zhao Y Xiong H Sneh T Fan Y. Complement and Coagulation Cascades Pathway Correlates With Chemosensitivity and Overall Survival in Patients With Soft Tissue Sarcoma. Eur J Pharmacol (2020) 879:173121. doi: 10.1016/j.ejphar.2020.173121 32339514

[33] Guo Z Wang F Di Y Yao L Yu X Fu D. Antitumor Effect of Gemcitabine-Loaded Albumin Nanoparticle on Gemcitabine-Resistant Pancreatic Cancer Induced by Low Hent1 Expression. Int J Nanomedicine (2018) 13:4869–80. doi: 10.2147/IJN.S166769 PMC6122898 30214194

[34] Sorolla A Wang E Golden E Duffy C Henriques ST Redfern AD. Precision Medicine by Designer Interference Peptides: Applications in Oncology and Molecular Therapeutics. Oncogene (2020) 39(6):1167–84. doi: 10.1038/s41388-019-1056-3 PMC7002299 31636382

[35] Rodríguez-Tomàs E Murcia M Arenas M Arguís M Gil M Amigó N. Serum Paraoxonase-1-Related Variables and Lipoprotein Profile in Patients With Lung or Head and Neck Cancer: Effect of Radiotherapy. Antioxidants (Basel) (2019) 8(7):213. doi: 10.3390/antiox8070213 PMC6680864 31295833

[36] Ding GY Zhu XD Ji Y Shi GM Shen YH Zhou J. Serum PON1 as a Biomarker for the Estimation of Microvascular Invasion in Hepatocellular Carcinoma. Ann Transl Med (2020) 8(5):204. doi: 10.21037/atm.2020.01.44 32309351 PMC7154400

[37] Marsillach J Camps J Ferré N Beltran R Rull A Mackness B. Paraoxonase-1 Is Related to Inflammation, Fibrosis and PPAR Delta in Experimental Liver Disease. BMC Gastroenterol (2009) 9:3. doi: 10.1186/1471-230X-9-3 19144177 PMC2632645

[38] Bao L Liu H You B Gu M Shi S Shan Y. Overexpression of IGFBP3 is Associated With Poor Prognosis and Tumor Metastasis in Nasopharyngeal Carcinoma. Tumour Biol (2016) 37(11):15043–52. doi: 10.1007/s13277-016-5400-8 27658775

[39] Yin L Chen J Ma C Pei S Du M Zhang Y. Hsa_circ_0046263 Functions as a ceRNA to Promote Nasopharyngeal Carcinoma Progression by Upregulating IGFBP3. Cell Death Dis (2020) 11(7):562. doi: 10.1038/s41419-020-02785-3 32703944 PMC7378203

[40] Ranke MB. Insulin-Like Growth Factor Binding-Protein-3 (IGFBP-3). Best Pract Res Clin Endocrinol Metab (2015) 29(5):701–11. doi: 10.1016/j.beem.2015.06.003 26522455

[41] Guan S Wei J Huang L Wu L. Chemotherapy and Chemo-Resistance in Nasopharyngeal Carcinoma. Eur J Med Chem (2020) 207:112758. doi: 10.1016/j.ejmech.2020.112758 32858472

[42] Cui Y Hunt A Li Z Birkin E Lane J Ruge F. Lead DEAD/H Box Helicase Biomarkers With the Therapeutic Potential Identified by Integrated Bioinformatic Approaches in Lung Cancer. Comput Struct Biotechnol J (2020) 19:261–78. doi: 10.1016/j.csbj.2020.12.007 PMC7779375 33425256

[43] Yu B Liang H Ye Q Wang Y. Establishment of a Genomic-Clinicopathologic Nomogram for Predicting Early Recurrence of Hepatocellular Carcinoma After R0 Resection. J Gastrointest Surg (2021) 25(1):112–24. doi: 10.1007/s11605-020-04554-1 32128678

[44] Tlemsani C Pongor L Elloumi F Girard L Huffman KE Roper N. SCLC-CellMiner: A Resource for Small Cell Lung Cancer Cell Line Genomics and Pharmacology Based on Genomic Signatures. Cell Rep (2020) 33(3):108296. doi: 10.1016/j.celrep.2020.108296 33086069 PMC7643325

[45] Pogrebniak KL Curtis C. Harnessing Tumor Evolution to Circumvent Resistance. Trends Genet (2018) 34(8):639–51. doi: 10.1016/j.tig.2018.05.007 PMC6368975 29903534

