# Supplementary material for: Novel Plasma Proteomic Biomarkers for Early Identification of Induction Chemotherapy Beneficiaries in Locoregionally Advanced Nasopharyngeal Carcinoma
Source: Front Oncol. 2022 Jun 30;12:889516. doi: 10.3389/fonc.2022.889516 (PMC9279567; doi:10.3389/fonc.2022.889516)
Supplement: Supplementary file 1 [file DataSheet_1.docx]

Supplementary Material

# Supplementary Figure and Table

## Supplementary Figure


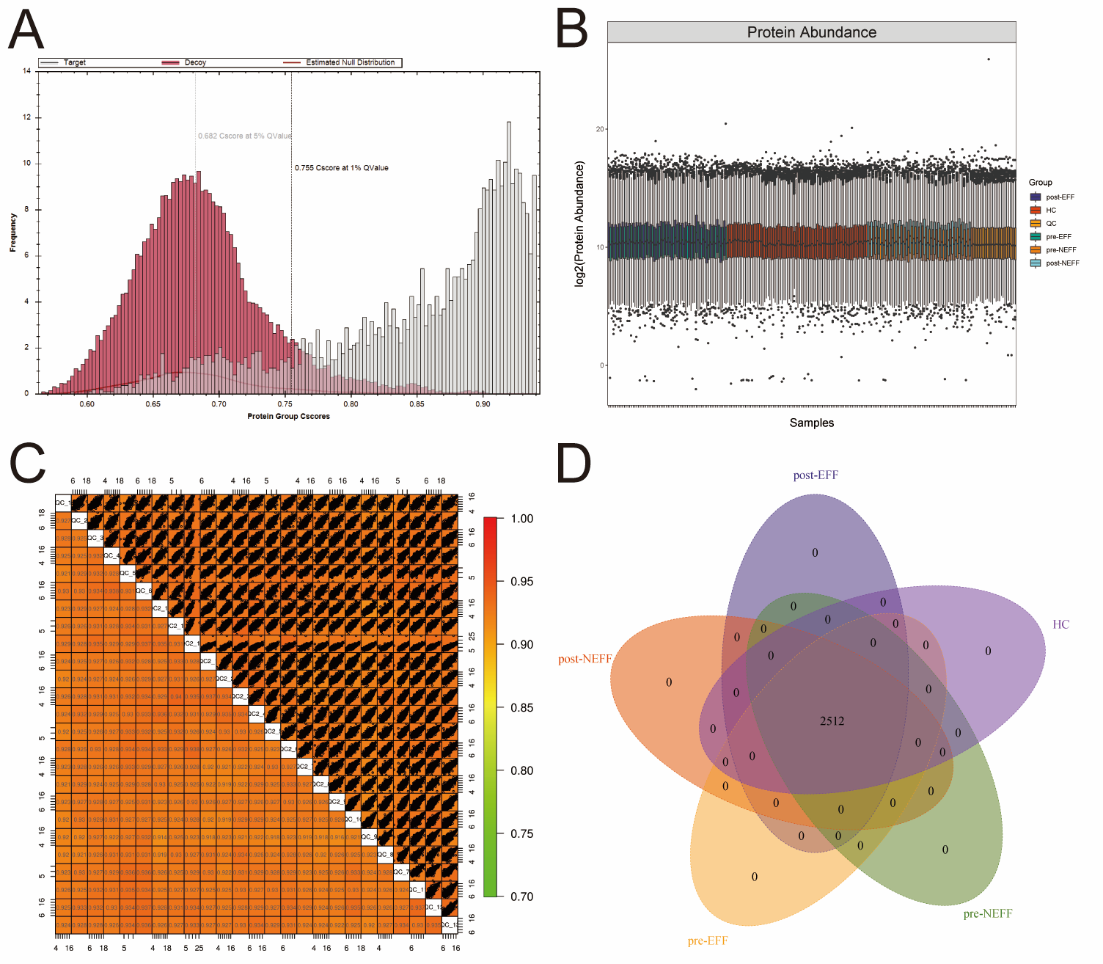


**Supplementary Figure 1.** **Quality control of DIA**
**(A)** Protein FDR distribution plot. The abscissa represents the Cscore values of proteins, and the ordinate represents the number of proteins below a certain Cscore. The black line is the 1% Q value standard line. Red indicates the decoy and grey indicates the target. **(B)** Intensity jitter distribution plot of sample quantitation. The abscissa represents the sample and the ordinate represents protein expression. Different colours represent different groups. **(C)** Strength correlation heatmap of quality control samples. The abscissa and ordinate represent the logarithm of the strength value. **(D)** Venn diagram showing the overlap of proteomic profiles among HC, pre-EFF, pre-NEFF, post-EFF and post-NEFF groups.

## Supplementary Table

**Supplementary Table 1.** **GTV changes before and after IC in patients with LA-NPC**

| **Cohort** | **ID** | **GTV (mm^3^)** | | **Response** |
| --- | --- | --- | --- | --- |
|  |  | **Pre-IC** | **Post-IC** |  |
| EFF | 01 | 32.41 | 18.03 | PR |
|  | 02 | 41.12 | 23.53 | PR |
|  | 03 | 25.16 | 11.91 | PR |
|  | 04 | 11.95 | 8.44 | PR |
|  | 05 | 15.27 | 9.16 | PR |
|  | 06 | 12.28 | 6.91 | PR |
|  | 07 | 14.41 | 9.25 | PR |
|  | 08 | 10.11 | 5.35 | PR |
|  | 09 | 25.96 | 13.87 | PR |
|  | 10 | 21.02 | 12.45 | PR |
|  | 11 | 31.70 | 20.99 | PR |
|  | 12 | 27.71 | 19.73 | PR |
|  | 13 | 17.46 | 12.51 | PR |
|  | 14 | 26.92 | 13.98 | PR |
|  | 15 | 29.84 | 12.65 | PR |
|  | 16 | 21.26 | 11.33 | PR |
|  | 17 | 16.77 | 12.14 | PR |
|  | 18 | 16.04 | 10.98 | PR |
|  | 19 | 10.48 | 5.84 | PR |
|  | 20 | 13.51 | 8.43 | PR |
|  | 21 | 24.17 | 14.99 | PR |
|  | 22 | 20.58 | 14.27 | PR |
|  | 23 | 41.28 | 12.02 | PR |
|  | 24 | 21.37 | 17.78 | PR |
|  | 25 | 5.19 | 3.43 | PR |
|  | 26 | 7.83 | - | CR |
|  | 27 | 21.96 | 12.61 | PR |
|  | 28 | 13.3 | 9.8 | PR |
|  | 29 | 30.08 | 19.67 | PR |
|  | 30 | 28.37 | 16.51 | PR |
|  | 31 | 33.61 | 17.48 | PR |
|  | 32 | 13.56 | 9.81 | PR |
|  | 33 | 23.72 | 12.88 | PR |
|  | 34 | 19.21 | 11.83 | PR |
| NEFF | 01 | 43.50 | 64.83 | PD |
|  | 02 | 6.43 | 7.92 | SD |
|  | 03 | 29.75 | 26.36 | SD |
|  | 04 | 5.17 | 5.17 | SD |
|  | 05 | 12.87 | 15.89 | SD |
|  | 06 | 20.79 | 19.30 | SD |
|  | 08 | 8.14 | 6.61 | SD |
|  | 09 | 51.97 | 45.92 | SD |
|  | 10 | 34.54 | 35.51 | SD |
|  | 11 | 24.36 | 24.66 | SD |
|  | 12 | 15.98 | 13.08 | SD |
|  | 13 | 105.92 | 113.91 | SD |
|  | 14 | 21.34 | 18.46 | SD |
|  | 15 | 12.27 | 10.83 | SD |
|  | 16 | 23.90 | 22.73 | SD |
|  | 17 | 11.28 | 9.31 | SD |
|  | 18 | 14.15 | 13.79 | SD |
|  | 19 | 28.34 | 23.63 | SD |
|  | 20 | 86.19 | 78.97 | SD |
|  | 21 | 15.51 | 19.80 | SD |
|  | 22 | 22.57 | 19.62 | SD |
|  | 23 | 45.83 | 42.31 | SD |
|  | 24 | 53.36 | 48.74 | SD |
|  | 25 | 28.49 | 25.68 | SD |
|  | 26 | 15.64 | 16.22 | SD |
|  | 27 | 28.89 | 29.42 | SD |
|  | 28 | 40.81 | 38.67 | SD |
|  | 29 | 32.17 | 29.58 | SD |
|  | 30 | 12.27 | 10.96 | SD |

Abbreviations: PR, partial response; CR, complete response; PD, progressive disease; SD, stable disease.
